# Understanding the impact of growth hormone on ventilatory control stability in children with Prader-Willi syndrome

**DOI:** 10.1007/s00431-026-06857-y

**Published:** 2026-03-20

**Authors:** Okkes R. Patoglu, Rosemary S. C. Horne, Dwayne L. Mann, Shane A. Landry, Nitin Kapur, Samara Thambar, Jacob Robinson, Margot J. Davey, Gillian M. Nixon, Bradley A. Edwards

**Affiliations:** 1 Department of Paediatrics, Monash University, Melbourne, VIC Australia; 2 School of Electrical Engineering and Computer Science, The University of Queensland, Brisbane, QLD Australia; 3 School of Psychological Sciences, Monash University, Melbourne, VIC Australia; 4 Department of Paediatric Respiratory and Sleep Medicine, Queensland Children’s Hospital, Brisbane, QLD Australia; 5 Children’s Health Queensland Clinical Unit, Faculty of Medicine, The University of Queensland, Brisbane, QLD Australia; 6 Melbourne Children’s Sleep Centre, Monash Children’s Hospital, Melbourne, VIC Australia

**Keywords:** Prader-Willi syndrome, Paediatric, Sleep, Ventilatory control, Loop gain

## Abstract

A hallmark of Prader-Willi syndrome (PWS) is hypothalamic-pituitary axis dysfunction, which can result in reduced growth hormone (GH) production. While GH replacement therapy is common in children with PWS, it has also been implicated in the development of obstructive sleep apnoea (OSA) in some children. The mechanisms underlying this development are poorly understood but may be related to alterations in ventilatory control. Our study investigated the impact of GH treatment on ventilatory control stability during sleep in children with PWS.

Polysomnographic data pre- and post-GH therapy in 25 children (aged 2mo-18y) were used to assess ventilatory control using a validated method that estimates loop gain (dimensionless ratio) from ventilation changes following spontaneous sighs during sleep. Data were analysed using linear mixed-effects modelling with GH as a fixed effect and participant as a random intercept. Covariates that could impact loop gain including age, obstructive-apnoea hypopnoea index (OAHI) and central apnoea-hypopnoea index (CAHI) were each added separately to the base model in a stepwise, manual forward selection approach.

Loop gain was not altered by GH treatment (*β* = 0.003, 95% CI: [-0.042, 0.049], *p* = 0.878, Cohen’s d = 0.031). Age, OAHI and CAHI did not alter the impact of GH on loop gain. No difference in sleep or respiratory characteristics were found, however 20% of children developed OSA post-GH.

*Conclusion*: Initiation of GH therapy was not associated with a change in loop gain, suggesting that changes in ventilatory control are unlikely to contribute to the development of OSA in children with PWS.

**Table:** 

**What is Known:** *• Prader-Willi syndrome is associated with abnormal ventilatory control and increased risk of sleep-disordered breathing.* *• Growth hormone therapy may influence respiratory physiology but its effect on the stability of ventilatory control (loop gain) remains unclear.*	
**What is New:** *• In this cohort of children with Prader-Willi syndrome, growth hormone therapy did not alter loop gain despite inter-individual variability.* *• Our findings suggest that any sleep-disordered breathing that emerges following growth hormone therapy is likely driven by mechanisms other than altered loop gain.*

**Supplementary Information:**

The online version contains supplementary material available at 10.1007/s00431-026-06857-y.

## Introduction

Prader-Willi syndrome (PWS) is a neurodevelopmental disorder caused by the absence of expression of paternally inherited genes on chromosome 15q11.2–q13 [1]. It is characterised by a range of physiological, behavioural, endocrine [2] and autonomic nervous system abnormalities [3]. However, a hallmark feature is dysfunction in the hypothalamic-pituitary axis [4], resulting in reduced growth hormone (GH) secretion in some children. Therefore, children with PWS often receive GH replacement therapy early in life [5] as it has been demonstrated to improve body composition, motor development, and quality of life [5, 6]. Despite these benefits, GH has been associated with heterogenous and unpredictable changes in sleep-disordered breathing in individuals with PWS, with both obstructive and central sleep apnoea (OSA and CSA respectively) variably improving, worsening, or remaining unchanged following treatment [7–13]. Even in the absence of GH treatment, children with PWS have a higher prevalence of OSA [14, 15] and CSA [16] compared to typically developing children, suggesting underlying pathophysiological predispositions in PWS that increase their susceptibility.

It is well established that OSA is multifactorial in both adults [17] and children [18], comprising both anatomical (i.e., adenotonsillar hypertrophy) and non-anatomical contributors [17, 18] such as abnormal ventilatory control. In children with PWS, the increased prevalence of OSA is likely due to the combination of anatomical compromise (craniofacial features, hypotonia etc.) but also potentially decreased chemosensitivity (and the ensuing upper airway muscle hypotonia due to low drive) which may further influence the propensity towards airway collapse [14, 15]. Both adults and children with PWS have been demonstrated to have blunted hypercapnic ventilatory responses [14, 19–21], indicating reduced chemosensitivity to carbon dioxide. Furthermore in one study 92% of children with PWS failed to arouse in response to hypoxic challenge [22], supporting the notion PWS may be associated with impaired chemoreceptor and arousal control mechanisms.

Studies that have examined the impact of GH for its potential to modulate ventilatory control in children with PWS have shown mixed findings. One investigation found an increased hypercapnic ventilatory response and improved resting ventilation after GH treatment [23]. Another study finding no difference in the hypercapnic ventilatory response during sleep after GH [24]. There are two major differences between these studies which may explain the contrasting findings: the age of the children and the state in which the investigations were conducted. The study by Katz-Salamon et al., studied younger children aged 0.3–5.3 years during sleep [24], while Lindgren et al., studied older children aged 7–14 years during wakefulness [23]. A key limitation of both studies was that their investigations focused solely on one component of the ventilatory control system, chemosensitivity. Ventilatory control during sleep is regulated by a negative feedback loop comprised of three main components: (1) chemosensitivity (controller gain), (2) lung/body gas exchange dynamics (plant gain) and (3) the circulatory delay, which are collectively quantified by loop gain, a measure of the overall stability of ventilatory control. Thus, the entire negative feedback loop needs investigation in order to gain a more comprehensive understanding of how GH may impact ventilatory control. The aim of the current study was therefore to assess the impact of GH treatment on loop gain in children with PWS to yield important insights into the pathophysiological mechanisms underlying sleep-disordered breathing in this at-risk population.

## Methods

Ethical approval for this study was granted by Monash Health Human Research Ethics Committee (RES-22–0000-035L) and parents/guardians provided informed written consent before the study and was in accordance with the National Health and Medical Research Council Act 1992 and the National Statement on Ethical Conduct in Human Research (2018). This retrospective, observational study included children with PWS aged < 18 years who underwent in-hospital polysomnography (PSG) both prior to GH initiation and within 12 months of commencing GH therapy between December 2010 and December 2023 at either Monash Children’s Hospital in Melbourne, or Queensland Children’s Hospital in Brisbane, Australia. All children were well at the time of their PSG studies, including being free of symptoms of an upper respiratory tract infection. For children who had multiple PSGs, the study closest in timing before GH initiation was chosen as the baseline study, and the first PSG after commencing GH was selected as the post-GH study. Because oxygen therapy is known to impact ventilatory control [25–27], if supplemental oxygen was used for part of the PSG, only the portion of the PSG without oxygen therapy was included in the current analysis. Height and weight were measured at the PSG visit and body mass index (BMI) Z-score calculated for children > 2 years of age [28].

### Polysomnographic recordings

Overnight attended PSG studies were conducted and manually scored by paediatric sleep scientists in compliance with guidelines established by the American Academy of Sleep Medicine at the time of the study [29–32]. For studies recorded prior to 2012, NREM 3 and NREM 4 were combined into N3 to be consistent with current staging rules. Studies scored using infant criteria as active (AS) and quiet sleep (QS) [33] were included in the sleep state analysis as REM and NREM respectively, without sub-categorisation of NREM sleep. The obstructive apnoea-hypopnoea index (OAHI), defined as the total number of obstructive apnoeas and hypopnoeas, and mixed apnoeas per hour of total sleep time (TST) was used to define OSA severity. An OAHI of ≤ 1 event/h defined primary snoring, > 1 < 5 events/h mild OSA, ≥ 5 < 10 events/h as moderate OSA and ≥ 10 events/h as severe OSA [34]. A central apnoea-hypopnoea index (CAHI), defined as the total number of central apnoeas and hypopnoeas per hour of TST, of > 5 events/h was used to define presence of CSA [35–37]. The respiratory disturbance index (RDI) was defined as the total number of obstructive, central and mixed apnoeas and hypopnoeas per hour of sleep.

### Assessment of loop gain

PSG data (Compumedics Ltd, Abbotsford, Australia) were exported to European Data Format and imported into MATLAB (MATLAB R2018a, MathWorks, Natick, MA, USA) to quantify loop gain. For this analysis, we assessed the ventilatory response that follows spontaneous sighs during sleep as described previously [38–41]. Only PSGs with good quality nasal pressure and respiratory bands were used to visually identify movement/artifact free spontaneous sigh breaths during both NREM and REM sleep. To be included in the analysis, at least 3 analysable spontaneous sighs in each of the pre- and post-GH studies were required [38]. For each sigh identified, a 180 s analysis window was placed around each sigh, 60 s before and 120 s after the sigh breath. Quantification of loop gain was derived by fitting a validated model of ventilatory control (comprising gain, time-constant and delay of the negative feedback loop) to the pre- and post-sigh ventilatory patterns [42, 43]. In this model, gain represents overall loop gain, time-constant reflects the time to buffer CO_2_ in the lung and tissues, and delay reflects the circulatory time between the lung and chemoreceptors. The primary outcome was the loop gain measured at the natural cycling frequency (i.e., LGn). As a sensitivity analysis we also investigated loop gain at a frequency of 4 cycles/minute (LG4) and 1 cycle/minute (LG1), presented in Supplementary Material. In addition to the primary outcome, we also investigated the impact of GH on (1) Tn, the natural period of the ventilatory control system reflecting the intrinsic length of its oscillatory cycle, (2) sigh magnitude (%V_eupnea_), representing the percentage of resting ventilation, (3) Tau, the time constant that describes how quickly the chemical drive for breathing builds up in response to changes in ventilation, and (4) Delay, the circulatory latency between lung ventilation and chemoreceptors. Each analysis window estimated each of the listed parameters above, which were then averaged to produce a single value per individual and condition (pre- and post-GH).

### Statistical analysis

Statistical analyses were carried out using R software (version 4.5.0, R Foundation for Statistical Computing, Vienna, Austria). Normality was determined both visually using Q-Q plots and using the Shapiro–Wilk test. Demographic, sleep and respiratory characteristics were compared with the paired Student’s t-test for normally distributed data and the Wilcoxon signed rank test for non-normally distributed data. As the majority of the demographic and respiratory data were not normally distributed, summary statistics are presented as median and interquartile range (IQR) irrespective of normality for consistency and ease of interpretation. Baseline characteristics between the included children and those excluded due to inadequate nasal pressure signals were compared with the Mann–Whitney U tests for continuous variables and the Chi-square test for sex. Linear mixed-effects models with a random intercept for participant were used to examine the effect of GH on loop gain (and other outcome variables), with effects quantified using the beta coefficient (*β*) and 95% confidence interval (CI). Cohen’s d was computed for the main outcome as the mean paired difference divided by the standard deviation of the paired differences to provide an estimate of the magnitude of change in standard deviation units for ease of interpretation. Models were fitted with and without the covariates of age, OAHI and CAHI. Simple linear regression analyses were performed to examine the association between the change (post-GH—baseline) in loop gain (∆LGn) and the change in OAHI (∆OAHI), and between the ∆OAHI and the change in sigh magnitude. Assumptions of linear regression models were assessed using standardised residuals. Normality of residuals was evaluated via inspection of Q-Q plots, while homoscedasticity and overall model fit were assessed using residual-versus-fitted plots and coefficients of determination (*R*^2^). Outliers were identified using two criteria: (1) y-outliers defined as observations with absolute standardised residuals > 3, and (2) influential points, identified using Cook’s distance > 4/n. An adjusted model was then fit excluding outliers and model assumptions were reassessed. A p-value of < 0.05 was considered statistically significant.

## Results

**Fig. 1:**
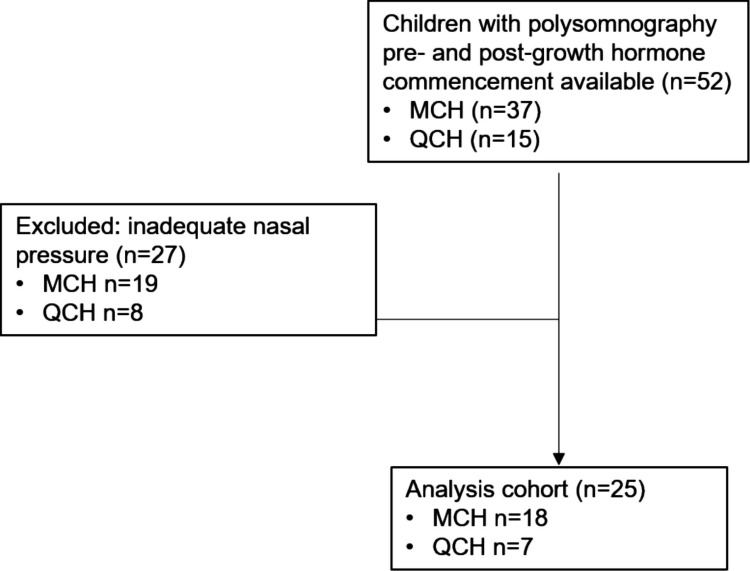
Flow diagram of participant recruitment at the Melbourne Children’s Hospital (MCH) and Queensland Children’s Hospital (QCH)

Of the 52 children with PWS identified with a PSG pre- and post-GH in the study period, 27 did not have adequate nasal pressure signals; these children were excluded, leaving 25 children included in the final analysis (Fig. 1). The children’s age ranged from 2–149 months with an average time between studies of 6.4 months (range 2–12 months). Four children at baseline and one child post-GH had supplemental oxygen administration during a portion of their PSG; only PSG data from the portion without supplemental oxygen was analysed. One infant’s sleep was scored as AS and QS and was included in the analysis. The 27 children excluded were not different in terms of age, BMI, OAHI or CAHI to the children included (*n* = 25) in the analysis (data not shown).

### Demographic sleep and respiratory characteristics

Demographic, sleep and respiratory characteristics of the included children are compared between the baseline and post-GH studies in Table 1. No differences in any sleep or respiratory characteristics were found. Although there was no significant difference in OAHI or CAHI after GH, 5 children (20%) developed new OSA during GH therapy (4 mild, 1 moderate). These comprised two infants, two children aged 1–3 years, and one aged 7.8 years at baseline. By contrast, 2 (8%) children had decreased OSA severity from mild OSA to primary snoring and 1 (4%) from moderate OSA to mild OSA.

**Table 1:** Demographic, sleep and respiratory characteristics of children with Prader-Willi syndrome Pre- and Post-growth hormone (GH)

	Pre-GH	Post-GH	*p*-value
n	25	25	
Sex	13 F (52%)	13 F (52%)	
Age (months)	14.0 (8.0, 68.0)	22.0 (15.5, 78.0)	< 0.001
BMI Z-score*	1.5 (0.4, 2.5)	1.2 (1.1, 2.4)	0.35
Total Sleep Time (min)	472.5 (450.5, 500.0)	477.5 (455.0, 498.5)	0.83
Sleep Latency (min)	9.5 (0.5, 23.1)	10.5 (0.1, 19.0)	0.79
Wake after sleep onset (%)	9.5 (6.0, 11.8)	8.4 (4.5, 12.8)	0.76
Sleep efficiency %	88.0 (84.3, 91.0)	88.7 (85.6, 92.8)	1.00
NREM (%)	74.9 (65.5, 78.1)	77.0 (72.5, 78.7)	0.31
N1 (%)	5.9 (1.5, 11.9)	5.8 (1.4, 9.9)	0.50
N2 (%)	43.4 (34.3, 54.4)	49.0 (44.8, 53.1)	0.12
N3 (%)	23.5 (17.3, 27.0)	18.6 (16.8, 24.8)	0.21
REM (%)	24.7 (20.0, 30.6)	22.0 (21.3, 27.6)	0.59
OAHI (events/h)	0.4 (0.0, 0.5)	0.4 (0.0, 1.1)	0.52
CAHI (events/h)	4.4 (2.1, 7.1)	4.4 (2.5, 6.0)	0.36
RDI (events/h)	6.4 (3.0, 9.3)	4.9 (3.1, 7.4)	0.42
Arousal Index (events/h)	8.0 (6.6, 11.1)	7.3 (4.7, 11.5)	0.14
SpO_2_ nadir (%)	83.0 (76.0, 89.5)	84.0 (74.5, 88.0)	0.57

Data presented as median (first, third quartiles) or count (frequency)

*BMI Z-score  *standardised body mass index for age and sex*, NREM* non-rapid eye movement, *REM* rapid eye movement, *OAHI* obstructive apnoea-hypopnoea index, *CAHI* central apnoea-hypopnoea index, *RDI* respiratory disturbance index

*BMI Z-score data only available for *n* = 11 children

### Impact of Growth Hormone on loop gain and other model parameters

**Fig. 2:**
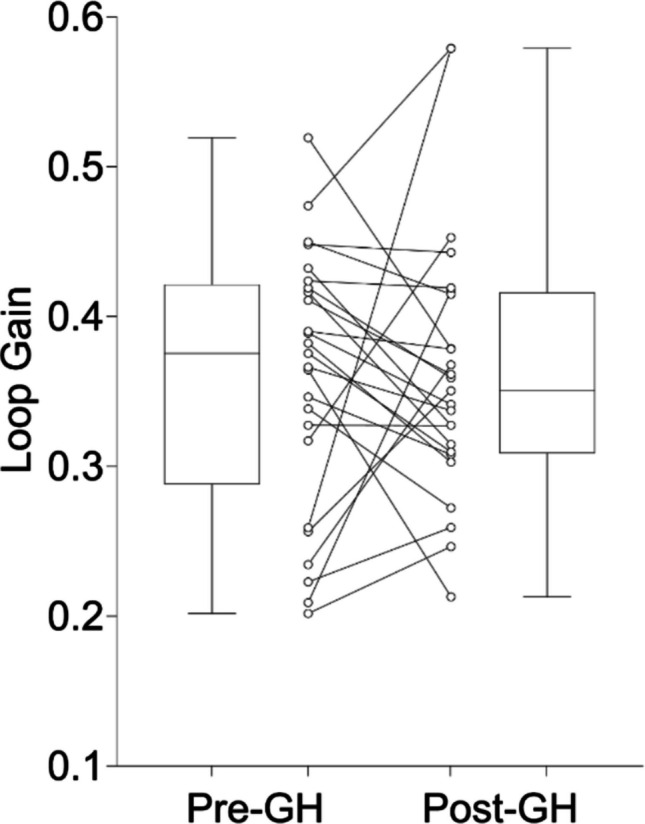
Average loop gain measured at a natural cycling frequency (LGn) for each child with Prader-Willi syndrome Pre- and Post-Growth Hormone (GH) treatment. Box represents the interquartile range (25th and 75th percentiles), horizontal line in the box represents the median, and the whiskers extend to minimum and maximum values. Individual data shown in the middle to illustrate the variable response observed

During total sleep, GH treatment did not significantly alter LGn (*β* = 0.003, 95% CI: [−0.042, 0.049], *p* = 0.878, Cohen’s d = 0.031), as shown in Fig. 2.

In step-wise mixed-effects models, age, OAHI and CAHI were added individually as covariates. Neither age, OAHI, or CAHI at baseline impacted the effect of GH on LGn (Table 2). However, independent of GH, age was related to LGn during total sleep, with each additional year of age associated with an increase in LGn of 0.008 units (*p* = 0.033). During NREM sleep, GH did not significantly alter LGn in the unadjusted model (*β* = 0.012, 95% CI: [−0.040, 0.064], *p* = 0.644) or when controlling for age, OAHI and CAHI. When the same investigation was conducted for LG1 and LG4, there were also no significant differences between pre- and post-GH conditions, including after adjusting for age, OAHI and CAHI, as well as when analysing NREM sleep only (see Supplementary Tables S1 and S2). To explore whether the impact of GH on LGn differed by sex, linear mixed-effects models were run on males and females separately. Similar to the main findings, there were no significant change in LGn following GH in either group (males: *β* = 0.014, 95% CI: [−0.089, 0.061], *p* = 0.689; females: *β* = −0.006, 95% CI: [−0.059, 0.072], *p* = 0.838).

**Table 2:** Impact of growth hormone on loop gain measured at a natural cycling frequency (LGn), as well as the impact of covariates in both total sleep and NREM sleep

	No Covariate **-**Original model	+ Age	+ OAHI	+ CAHI
Total Sleep	0.003 (−0.042, 0.049), *p* = 0.878	−0.001 (−0.047, 0.045), *p* = 0.965	0.002 (−0.045, 0.050), *p* = 0.921	0.004 (−0.042, 0.051), *p* = 0.845
NREM	0.012 (−0.040, 0.064), *p* = 0.644	0.008 (−0.044, 0.061), *p* = 0.742	0.010 (−0.043, 0.062), *p* = 0.712	0.013 (−0.040, 0.065, *p* = 0.629

Values represent the unstandardized LGn beta-estimates (95% confidence intervals) and *p*-values from linear mixed-effects models

*NREM* non-rapid eye movement, *OAHI* obstructive apnoea-hypopnoea index, *CAHI* central apnoea-hypopnoea index

While GH did not significantly impact several other model parameters (Tn, Tau and Delay), the sigh magnitude was modestly reduced post-GH (*β* = −0.201 (%V_eupnea_), 95% CI [−0.384, −0.017], *p* = 0.033). We therefore investigated whether change (∆) in sigh magnitude and LGn were related to change in OAHI. The ∆LGn was not significantly associated with the ∆OAHI (*R*^2^ = 0.029, *p* = 0.415). Interestingly, a weak yet significant negative association was observed between ∆sigh magnitude and the ∆OAHI (*R*^2^ = 0.208, *p* = 0.022). This relationship became even weaker in a sensitivity analysis excluding outliers (*R*^2^ = 0.134, *p* = 0.079).

## Discussion

This is the first study to investigate the impact of GH on the sensitivity of the negative feedback loop that controls ventilation (i.e. loop gain) in children with PWS. Amongst the 25 children included in our analysis, we found that GH therapy did not significantly impact loop gain in the first months of treatment, with no difference observed between sexes. Our analyses also showed that GH was associated with a modest reduction in the sigh magnitude, whereas the other model parameters (Tn, Tau and Delay) were unaffected. Demographic, sleep and respiratory characteristics were also unchanged, with no overall differences in either OAHI or CAHI post-GH. While the change in the OAHI post-GH treatment was not related to the change in loop gain, it was partly explained by the change in the magnitude of the spontaneous sighs.

### The effects of GH on ventilatory control

Previous, albeit limited, evidence suggests that the increased prevalence of OSA and CSA seen in individuals with PWS may be partly explained by impaired ventilatory control, characterised by blunted ventilatory responses to hypercapnia and hypoxia [14, 19, 20, 23]. The evidence of the impact of GH on ventilatory control in children with PWS is scarce, with only two studies examining the impact of GH, with conflicting findings. One investigation in 9 children aged 7–14 years reported an increased hypercapnic ventilatory response and approximately 26% improvement in resting ventilation during wakefulness after 6–9 months of GH therapy [23], suggesting an increase in controller gain. If controller gain does increase following GH, as suggested by Lindgren et al., then plant gain would need to be reduced by a similar degree in order to yield no significant change in loop gain as observed in our study. Given plant gain decreases with higher lung volume, improvements in stature, body composition, lean mass and BMI [44] or somatic growth [45] after GH therapy could plausibly explain such a change. By contrast, another investigation in 16 children aged 0.3–5.3 years found no difference in cardio-respiratory responses to oxygen and carbon dioxide during sleep after approximately 6 months of GH therapy [24]. The study by Katz-Salamon et al., reported that there were modest improvements in nocturnal oxygenation after GH, without a significant change in ventilatory responses to hyperoxia or hypercarbia, concluding that GH did not increase the chemoreceptor-mediated ventilatory response [24]. A major consideration in comparing our findings to the previous literature is the state (wakefulness vs sleep) of the children in the investigations. The control of ventilation and ventilatory responses to blood gases is reduced at sleep onset [46] and is sleep state dependent [47]. As such, the increase in controller gain found by Lindgren et al., during wakefulness, and the impact of GH, may not carry over into sleep. This notion is supported by the consistency with our findings and the findings by Katz-Salamon et al., where no impact of GH on ventilatory control during sleep was found. Notwithstanding, our study investigated the sensitivity of the overall feedback loop governing ventilation rather than focussing on controller gain only. Due to this, if GH does not impact controller gain as suggested by our findings and by Katz-Salamon et al., then there would be no change in plant gain to yield no net change in loop gain.

### Effect of GH on sigh magnitude and ventilatory function

We observed a modest reduction in sigh magnitude following GH treatment which may reflect improvement in ventilatory function by lowering plant gain. For example, in adults with childhood-onset GH deficiency, 12 months of GH therapy improved lung volumes and respiratory muscle strength [48]. Furthermore, GH therapy has been found to improve maximal oxygen uptake which may also reflect improved respiratory muscle strength [49]. During normal breathing, it is hypothesised that spontaneous sighs are a natural mechanism to re-expand alveoli to reverse any alveolar collapse [50]. Our findings may reflect a physiological adaptive response, whereby children with reduced lung function, as seen in GH deficiency, exhibit larger sigh amplitudes as a compensatory mechanism to re-expand alveoli. Following GH therapy, improvements in lung volume/function may reduce the need for this compensatory mechanism, resulting in a smaller sigh magnitude. While this finding is novel and speculative, it suggests that GH may not change the overall ventilatory control stability but may subtly enhance lung function and breathing mechanics, however further investigation is needed to validate this observation.

### Growth hormone, adenotonsillar hypertrophy, and OSA risk

We found a weak association between the change in sigh magnitude and the change in OAHI. Specifically, a reduction in sigh magnitude was correlated with an increase in OAHI following GH. This reflects the interplay and complexity of anatomical and non-anatomical contributors of OSA. For example a key link to the development or worsening of OSA in some children with PWS is that GH has been linked to adenotonsillar hypertrophy [51] by accelerating the growth of lymphoid tissues [7, 52]. The reduction in sigh magnitude may reflect improvement in lung function/volume (lower plant gain) as mentioned, however, adenotonsillar hypertrophy may explain why we did not observe a reduction in OSA overall. Furthermore, findings from our previous study in typically developing children found those with larger tonsils had significantly lower loop gain when compared with children with smaller tonsils [39], adding to the complexity of studying the pathophysiology behind the development of OSA and the role of loop gain. Our findings of no difference in OAHI after GH is consistent with prior studies that have shown no difference in the mean OAHI following GH [8, 12]. However, a subset of children (20%) in our study experienced worsening of OSA, and when reported as a proportion of children developing OSA, Caudri et al., have described similar rates [9]. One important consideration is the overlap with the pre-school window during which adenotonsillar hypertrophy typically increases and may contribute to OSA risk independent of GH therapy. In our cohort, two of the five children who developed OSA were within this window; however, cases also occurred in infancy and at approximately 8 years of age, suggesting that factors beyond adenotonsillar growth are potentially involved. Importantly, other studies have reported improvements in OSA severity during GH therapy [7, 9, 10], underscoring the heterogeneity of treatment responses and the likelihood that multiple developmental, anatomical and physiological factors shape individual trajectories.

### Strengths and limitations

A key strength of our study is the sample size (*n* = 25), which is the largest to date investigating ventilatory control in individuals with PWS. In addition, we tightly controlled for age, OAHI, CAHI and BMI. Our study included a large age range of children, including a large portion of children < 2 years (*n* = 14 at baseline), consistent with the current practice of initiating GH in infancy. Several limitations should nonetheless be considered. First, as we are the first to investigate loop gain and the effects of GH therapy in children with PWS, no prior data were available to inform a priori power calculation, which may increase the risk of type II error. However, the mean change in loop gain following GH therapy was very small relative to the within-subject variability. Power calculations based on the observed effect size indicate that several thousand participants would be required to detect a group-level difference with 80% power at a significance level of 0.05. These estimates imply that, despite limited sample size, it is improbable that GH therapy induces a clinically meaningful change in loop gain. Second, the method used to calculate loop gain requires an adequate nasal pressure signal which meant that 27 children could not be included in our analyses. Although we demonstrate that baseline clinical characteristics were similar between included and excluded children, our study remains at risk of selection bias and this may limit generalisability. Third, the 25 children were recruited over a prolonged period (2010–2023) from two centres. Changes in clinical practice over time and potential differences between hospitals may have introduced unmeasured variability. However, adjusting the main mixed-effects model for study site did not alter the estimate (*β* = 0.003, 95% CI −0.042 to 0.049, *p* = 0.878), and study site itself was not independently associated with loop gain (*β* = 0.024, 95% CI −0.039 to 0.087, *p* = 0.444). These findings suggest that centre‑related differences were unlikely to have materially influenced the main results, though residual confounding cannot be fully excluded. Finally, the loop gain methodology does not allow the assessment of its constituent components (i.e., controller and plant gains), limiting our ability to precisely elucidate whether GH impacts the individual components of the negative feedback loop governing ventilation.

## Conclusions

Growth hormone does not appear to significantly affect loop gain, making it a less likely contributor to the development of OSA in a subset of children. Our findings indicate that age plays a small role in shaping ventilatory control pointing towards a maturational increase over time in children with PWS. Reduced sigh magnitude was associated with an increased OAHI after GH, highlighting the complex interplay between various factors affecting the development of OSA. Because the current practice of starting GH for PWS is in infancy, future longitudinal studies will be important to determine how GH, when initiated in infancy, influences the developmental trajectory of loop gain into childhood and whether the heterogeneity of OSA outcomes reflects differential effects on ventilatory control.

## Supplementary Information

Below is the link to the electronic supplementary material.

Supplementary file1 (DOCX 17 KB)

## Data Availability

The data that support the findings of this study are available on request from RSC Horne (email: Rosemary.horne@monash.edu). The data are not publicly available due to privacy/ethical restrictions.

## Abbreviations

AS: Active sleep
CAHI: Central apnoea-hypopnoea index
CI: Confidence interval
CSA: Central sleep apnoea
GH: Growth hormone
IQR: Interquartile range
LG1: Loop gain at a frequency of 1 cycle/minute
LG4: Loop gain at a frequency of 4 cycle/minute
LGn: Loop gain at the natural cycling frequency
MCH: Monash children’s hospital
N1: Stage 1 non-rapid eye movement sleep
N2: Stage 2 non-rapid eye movement sleep
N3: Stage 3 non-rapid eye movement sleep
NREM: Non-rapid eye movement sleep
OAHI: Obstructive apnoea-hypopnoea index
OSA: Obstructive sleep apnoea
PSG: Polysomnography
PWS: Prader-Willi syndrome
QCH: Queensland children’s hospital
QS: Quiet sleep
RDI: Respiratory disturbance index
REM: Rapid eye movement sleep
TST: Total sleep time
