# Supplementary material for: Understanding the impact of growth hormone on ventilatory control stability in children with Prader-Willi syndrome
Source: Eur J Pediatr. 2026 Mar 20;185(4):194. doi: 10.1007/s00431-026-06857-y (PMC13002677; doi:10.1007/s00431-026-06857-y)
Supplement: Supplementary file 1 — Supplementary file1 (DOCX 17 KB) [file 431_2026_6857_MOESM1_ESM.docx]

**SUPPLEMENTAL MATERIAL**

**Table S1:** Effect of growth hormone on loop gain at a frequency of 1 cycle/minute (LG1) during total sleep and NREM sleep, presenting the estimated beta coefficient, 95% confidence interval, and p-value from linear mixed-effects models with and without covariate adjustment for age, obstructive apnoea-hypopnoea index (OAHI) and central apnoea-hypopnoea index (CAHI).

|  | **No Covariate** | **Age Covariate** | **OAHI Covariate** | **CAHI Covariate** |
| --- | --- | --- | --- | --- |
| **Total sleep** | -0.020,  (-0.141, 0.100), p=0.732 | -0.019,  (-0.140, 0.102), p=0.751 | -0.025,  (-0.152, 0.102), p=0.686 | -0.014,  (-0.135, 0.107), p=0.812 |
| **NREM** | -0.007,  (-0.140, 0.127), p=0.917 | -0.004,  (-0.137, 0.130), p=0.954 | -0.014,  (-0.146, 0.119), p=0.836 | 0.000,  (-0.134, 0.134), p=0.996 |

**Table S2:** Effect of growth hormone on loop gain at a frequency of 4 cycle/minute (LG4) during total sleep and NREM sleep, presenting the estimated beta coefficient, 95% confidence interval, and p-value from linear mixed-effects models with and without covariate adjustment for age, obstructive apnoea-hypopnoea index (OAHI) and central apnoea-hypopnoea index (CAHI).

|  | **No Covariate** | **Age Covariate** | **OAHI Covariate** | **CAHI Covariate** |
| --- | --- | --- | --- | --- |
| **Total sleep** | -0.004,  (-0.040, 0.031), p=0.804 | -0.003,  (-0.039, 0.033), p=0.864 | -0.005,  (-0.042, 0.032), p=0.780 | -0.002,  (-0.037, 0.034), p=0.925 |
| **NREM** | -0.001,  (-0.040, 0.037), p=0.941 | 0.001,  (-0.038, 0.040), p=0.977 | -0.002,  (-0.042, 0.038), p=0.915 | 0.001,  (-0.038, 0.040), p=0.946 |
